# A Comparison Study of Efficacy and Safety of a Biosimilar Form of Intramuscular Βeta-interferon I-a Versus the Reference Product: A Randomized Controlled Clinical Trial in Iran

**DOI:** 10.22037/ijpr.2019.14503.12441

**Published:** 2019

**Authors:** Seyed Massood Nabavi, Roya Abolfazli, Ali Etemadrezaei, Hamed Hosseini, Nahid Moradi, Sanaz Shahriari, Baharak Mehdipour, Babak Shekarchi, Akbar Soltanzadeh

**Affiliations:** a *Department of Brain and Cognitive Sciences, Royan Institute for Stem Cell Biology and Technology, Tehran, Iran.*; b *Department of Neurology, Amir Alam Hospital, Tehran University of Medical Sciences, Tehran, Iran. *; c *Actoverco Pharmaceuticals, Tehran, Iran. *; d *Clinical Trial Center, Tehran University of Medical Sciences, Tehran, Iran.*; e *Shefa Neuroscience Research Center, Tehran, Iran.*; f *Department of Radiology, School of Medicine, Aja University of Medical Sciences, Tehran, Iran*; g *Department of Neurology, Faculty of Medical, Tehran University of Medical Sciences, Tehran, Iran.*

**Keywords:** Relapsing remitting multiple sclerosis, Beta interferon -1a, Biosimilar, Multiple sclerosis, Pharmacologic therapy

## Abstract

We compared the efficacy and safety of a biosimilar form of beta-interferon-1a (Actovex) versus the reference product in the treatment of relapsing remitting multiple sclerosis (RRMS). In a double blind, randomized phase 3 clinical trial, we evaluated 138 patients with RRMS that were allocated to receive the biosimilar medication and the reference treatment (30 μg intramuscular, weekly for one year). We investigated changes in EDSS, relapse rate and MRI changes within one year. In sixty-nine patients who were allocated to each arm and analyzed mean age and its standard deviation was 32.4 ± 8.8 and 31.5 ± 8 for the biosimilar medication and the reference arm respectively. One-year follow-up revealed a mean difference of 0.084 in EDSS (95% CI: 0.069-0.237) between the two groups in favor of the biosimilar medication. This value did not exceed the predefined non-inferiority margin of 0.1. There were no statistically significant differences in relapse rate and systemic and local adverse events of the two groups. The results show that the biosimilar interferon 1-a is non-inferior to the reference product in terms of efficacy while it demonstrates comparable safety. In conclusion the biosimilar interferon 1-a can be considered as an effective and safe alternative to the reference product due to lower cost and more availability.

## Introduction

Multiple sclerosis is an inflammatory and autoimmune disease of the central nervous system and the relapsing remitting form is its most prevalent subtype. Despite the multiple therapeutic options for relapsing remitting multiple sclerosis (RRMS), interferons are still very commonly prescribed medications universally and in the Middle East (1-6). The highest prevalence of the disease is seen in the third or fourth decade of life with occurrence of one or more neural symptoms including sensory and motor, visual, brainstem, cerebellar, genitourinary tract, mood and digestion symptoms in patients. There may be partial recovery in the early stages of the disease, but progressive disability occurs over time (7).

Interferons cause reduction in annualized relapse rate (ARR) and slow the progression of disability. It has been shown that the progression of disability in patients treated with interferons ranges from 19 to 28%, which is similar to glatiramer acetate and fingolimod while, ARR is reduced 15-36% under treatment with interferons, glatiramer acetate (GA), and teriflunomide (8). 

Considering the role of interferons in treating RRMS and the ability of domestic pharmaceutical industry in producing these biologic products with lower cost, we designed this study to evaluate the safety and efficacy of Iranian Biosimilar Interferon beta-1a (Actovex) in comparison with the reference product in the Iranian patients diagnosed with RRMS in accordance to the regulations of Iranian food and drug administration.

## Experimental

In this double-blind randomized phase 3 clinical trial, 138 patients with RRMS were recruited to investigate the non-inferiority of the biosimilar interferon 1-a in comparison with the reference product from 1390 to 1395. This trial was registered at IRCT under the code IRCT2013020812398N1.

Eligibility criteria included age of 18 to 55 years, a definite RRMS diagnosis based on 2010 McDonald′s criteria, a history of at least 2 relapses over the last year, expanded disability status scale (EDSS) of 0 to 5.5, a wash-out period of 3 months if history of previous interferon or GA treatment existed.

The patients with progressive subtypes, history of recent recurrence (current or in the past 30 days) that resulted in at least 1 point increase in EDSS, any systemic disease or any organ dysfunction, history of hypersensitivity to interferons or other components of the drug, uncontrolled seizure, severe depression or the history of suicide in the previous three months, pregnancy, nursing, unwillingness to use a safe contraceptive method during the study, hypersensitivity or inability to perform MRI with gadolinium contrast, concomitant treatment with other immunomodulators, history of receiving cytotoxic drugs in the previous three months, history of receiving corticosteroid or other investigational drugs in the last month were excluded.

The patients were assigned to two arms with 4-block randomization scheme and were treated with 30 µg/1 mL/weekly of each drug for a year in identical manners.

The change in EDSS (primary outcome), severity and rate of relapses, percentage of relapse-free patients, radiologic findings (the number of Gadolinium enhancing lesions, enlarging, and new T2 lesions in MRI) represented efficacy outcomes, while flu-like symptoms, injection site reactions and laboratory measures represented safety outcomes for evaluation at 6 and 12-month intervals. 

We evaluated systemic adverse events (AEs) (headache, flu-like syndrome, fever, chills, and general pain) and local AEs (pain, erythema, itching, ecchymosis, and necrosis at the site of injection) during the study. 

In this study, non-inferiority margin was considered 0.1 differences of EDSS changes during one year follow up, which was estimated with Analysis of Covariance (for repeated observations). The analysis approach was per-protocol and missing data were replaced through multiple imputations by using linear regression.

## Results

**Figure 1:**
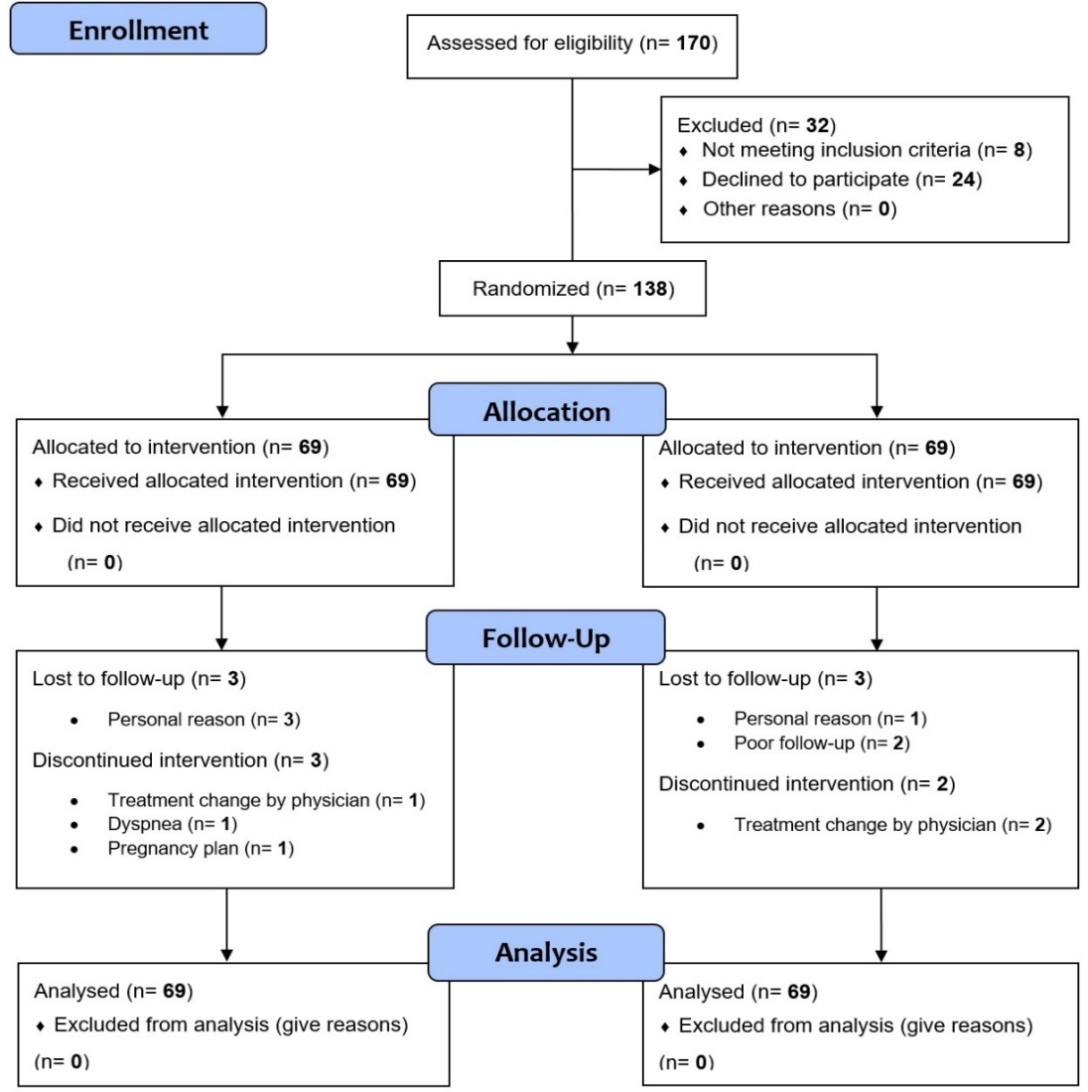
Disposition of patients throughout the study

One-hundred and seventy patients were evaluated for meeting the eligibility criteria of this study, out of which 8 did not meet the criteria and 24 refused to participate in the study. As a result, 138 patients (69 patients in each group) were randomized and divided into two arms of 69 patients each. The primary outcome endpoint was analyzed using intention-to-treat analysis containing all patients. The disposition of the patients throughout the study and the baseline characterisits of both arms are mentioned in Figure 1 and Table 1, respectively.

**Figure 2:**
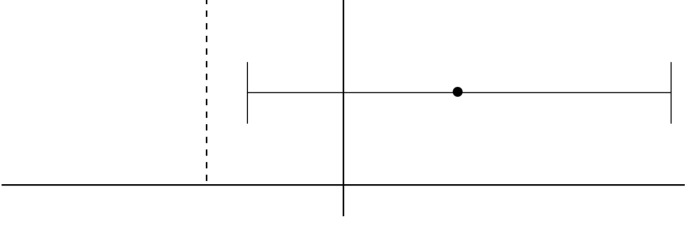
Estimation of difference of changes in EDSS in group Actovex versus Avonex

**Table 1:** Demographic and basic characteristics of the patients in two groups of Actovex and Avonex

**Variables**		**Group**	
	**Actovex (69)**	**Avonex (69)**	***P*** **-value**
Age (Mean ± SD)	32.48 (8.82)	31.51 (8.09)	0.502
Female	76.8% (53)	87.0% (60)	0.122
Family marriage	15.9% (11)	11.6% (8)	0.459
Family history	23.2% (16)	33.3% (23)	0.186
Currently employed	41.8% (28)	45.6% (31)	0.464
Diabetes Mellitus	2.9% (2)	1.4% (1)	0.559
Thyroid disease	11.6% (8)	8.7% (6)	0.573
Collagen-vascular disease	1.4% (1)	1.4% (1)	1.00
Hypertension	0	1.4% (1)	0.336
Liver disease	2.9% (2)	2.9 (2)	1.00
Renal disease	2.9% (2)	1.4% (1)	0.559
Migraine	10.1% (7)	21.7% (15)	0.063
Presenting symptoms			
Sensory	52.2% (36)	47.8% (33)	0.610
Motor	14.5% (10)	17.4% (12)	0.642
Brain stem	20.3% (14)	18.8% (13)	0.830
Cerebellar	21.7% (15)	13% (9)	0.178
Spinal	1.4% (1)	2.9% (2)	0.559
Visual	40.6% (28)	31.9% (22)	0.288
Sphincteric	4.3% (3)	0	0.080

**Table 2:** Comparison the side effects in first 6 months, second 6 months and one year

**Patients (n)**									
**Variable**		**First 6 month**			**Second 6 month**			**Total 1 year**	
	**Actovex (69)**	**Avonex (69)**	***p*** **-value**	**Actovex (64)**	**Avonex (63)**	***p*** **-value**	**Actovex (69)**	**Avonex (69)**	***p*** **-value**
Headache	68.1% (47)	79.7% (55)	0.121	43.8% (28)	69.8% (44)	0.003	71.0% (49)	84.1% (58)	0.066
Fever	65.2% (45)	78.3% (54)	0.089	28.1% (18)	41.3 (26)	0.120	71.0% (49)	79.7% (55)	0.236
Chills	65.2% (45)	68.1% (47)	0.718	29.7% (19)	44.4% (28)	0.085	71.0% (49)	72.5% (50)	0.850
General myalgia	78.3% (54)	85.5% (59)	0.269	60.9% (39)	73.0% (46)	0.148	81.2% (56)	85.5% (59)	0.493
Flu-like	59.4% (41)	73.9% (51)	0.071	21.9% (14)	41.3% (26)	0.019	63.8% (44)	75.4% (52)	0.139

**Table 3:** The mean serum levels of hepatobiliary function tests in both groups at baseline, month 6 and month 12

Serum level mean (SD)											
	**First 6 month**				**Second 6 months**				**Total 1 year**	***p*** **-value**
	**Actovex (69)**	**Avonex (68)**	***p*** **-value**	**Actovex (59)**	**Actovex (59)**	***p*** **-value**		**Actovex (57)**	**Avonex (59)**	
SGOT (AST)	17.86 (7.10)	15.85 (4.95)	0.058	16.81 (3.97)	18.37 (8.56)	0.208		17.35 (5.0.)	18.22 (8.21)	0.494
SGPT (ALT)	18.03 (7.90)	17.00 (9.73)	0.498	16.78 (7.00)	18.25 (9.03)	0.324		20.37 (12.00)	19.42 (10.20)	0.648
Alkaline phosphatase	152.28 (50.65)	150.46 (34.77)	0.807	146.56 (38.77)	152.12 (42.06)	0.455		156.54 (41.06)	146.10 (43.42)	0.186
Total Bilirubin	0.62 (0.36)	0.58 (0.31)	0.443	0.61 (0.27)	0.53 (0.30)	0.121	0.57		0.52	0.296

The results of this study showed that in the biosimilar group, the mean EDSS change (primary efficacy outcome) was 1.416 and 1.500 in the reference group, therefore, the EDSS of those in the biosimilar recipients was 0.084 lower than those with the other group (1.416; 95% CI: 1.308-1.525 in the biosimilar arm, 1.500; 95% CI: 1.392-1.608 in the reference arm) (Figure 2).

Seventy-two point five percent and eighty-one point five percent of the patients were relapse-free in Actovex and Avonex treatment arm, respectively (*p*-value = 0.23). In addition, the incidence of relapses and their severity during the first and second six months were not significantly different. The evaluation of MRI changes showed that the incidence of new T2 and GAD enhancing lesions were significantly lower in Actovex comparing with Avonex group both during the first 6 month and the 12 month evaluation.

During the study, no serious AEs were recorded. The incidence of headache and flu-like symptoms in the Actovex group were reportedly lower in the second six months in comparison with the reference group. There was also no significant difference between the two groups in terms of liver function tests (Tables 2 and 3).

## Discussion

This study aimed to investigate the non-inferiority of Actovex a biosimilar interferon to its reference product through a phase 3 clinical trial in treatment of RRMS. Non-inferiority analysis of the EDSS changes showed that in Actovex recipients, the score of disability was decreased 0.084 score more than other group during a year (Figure 2). Since this value is higher than the proposed non-inferiority margin of 0.1, non-inferiority of Actovex versus Avonex was proven. The EDSS changes in this trial in Avonex recipients were similar to the results in previous studies in Iranian patients in 2006 and 2012 (9, 10). We chose EDSS as the primary outcome measure in this trial to demonstrate efficacy of the treatment. EDSS was the primary outcome in the original trial that introduced Avonex as well. Investigating the incidence and severity of relapses showed no significant difference between the two groups. This finding was similarly reported in the Iranian population in the studies of Etemadifar and Naffisi and their colleagues as well (9, 10).

While there was no significant difference in the percentage of enlarging lesion between the two groups, we recorded significantly fewer new T2 and GAD enhancing lesions in Actovex treatment arm. Less radiologic activity of Actovex recipients may be explained by their slightly more advanced disease at baseline (Baseline EDSS 1.74 *vs.* 1.44) (11, 12).

Although, the safety measures between two groups were not significantly different, headache complaints and overall flu-like symptoms were significantly lower in Actovex treatment arm. This has a significant effect on long-term treatment and tolerability of the patient.

The prevalence of flu-like symptoms in this study was higher in comparison with previous Iranian studies (9, 10). The difference in the definition of these adverse events and its measurement method seems to be the main causes of this result. In the assessment of AEs (systemic, local and laboratory), there was no significant difference between the two groups and the results are similar to other studies. 

## Conclusion

The findings of this study show that the efficacy of Actovex is non-inferior to Avonex while safety and tolerability of the two drugs are comparable. This study emphasizes the lower cost and widespread availability of Actovex, a domestically manufactured medication, with comparable efficacy and safety to the reference product.

## References

[1] Compston A, Lassmann H, McDonald I (2006). The Story of Multiple Sclerosis. McAlpine’s Multiple Sclerosis.

[2] Deleu D, Mir D, Al Tabouki A, Mesraoua R, Mesraoua B, Akhtar N, Al Hail H, D′souza A, Melikyan G, Imam YZ, Osman Y, Elalamy O, Sokrab T, Kamran S, Ruiz Miyares F, Ibrahim F (2013). Prevalence, demographics and clinical characteristics of multiple sclerosis in Qatar. Mult. Scler. J.

[3] Pakdaman H, Amini Harandi A, Gharagozli K, Abbasi M, Tabassi A, Ashrafi F, Ghaffarpor M, Sharifi S, Delavar Kasmae H, Assarzadegan F, Arabahmadi M, Behnam B (2017). Health-related quality of life in patients with relapsing-remitting multiple sclerosis treated with subcutaneous interferon β-1a in Iran. Int. J. Neurosci.

[4] Yamout B, Barada W, Tohme RA, Mehio-Sibai A, Khalifeh R, El-Hajj T (2008). Clinical characteristics of multiple sclerosis in Lebanon. J. Neurol. Sci.

[5] Alroughani R, Ahmed SF, Al-Hashel J (2014). Demographics and clinical characteristics of multiple sclerosis in Kuwait. Eur. Neurol.

[6] Akhtar N, Elsetouhy A, Deleu D, Kamran S, Alhail H, Elalamy O, Mesraoua B, Sokrab T, Kamil H, Melikyan G, D′souza A, Osman Y, Imam Y (2013). Newly diagnosed multiple sclerosis in state of Qatar. Clin.Neurol. Neurosurg.

[7] Multiple sclerosis in adults: management (2014). National Institute for Health and Care Excellence.

[8] Kleinschnitz C, Niemczyk G, Rehberg-Weber K, Wernsdörfer C (2015). Interferon Beta-1a (AVONEX®) as a Treatment Option for Untreated Patients with Multiple Sclerosis (AXIOM): A Prospective, Observational Study. Int. J. Mol. Sci.

[9] Etemadifar M, Janghorbani M, Shaygannejad V (2006). Comparison of Betaferon, Avonex, and Rebif in treatment of relapsing–remitting multiple sclerosis. Acta. Neurol. Scand.

[10] Nafissi S, Azimi A, Amini-Harandi A, Salami S, Heshmat R (2012). Comparing efficacy and side effects of a weekly intramuscular biogeneric/biosimilar interferon beta-1a with Avonex in relapsing remitting multiple sclerosis: A double blind randomized clinical trial. Clin. Neurol. Neurosurg.

[11] Graziella Filippini L (2003). Interferons in relapsing remitting multiple sclerosis: a systematic review. Lancet.

[12] De Stefano N, Sormani MP, Stubinski B, Blevins G, Drulovic JS, Issard D, Shotekov P, Gasperini C (2012). Efficacy and safety of subcutaneous interferon beta-1a in relapsing–remitting multiple sclerosis: Further outcomes from the Improve study. J. Neurol. Sci..

[13] AVONEX® (interferon beta-1a) Intramuscular Injection Full Prescribing Information.

